# The interaction of study sequence presentation mode and response assignment reveals the effects of multiple computational systems on an immediate visual recognition task

**DOI:** 10.3758/s13414-025-03032-7

**Published:** 2025-02-26

**Authors:** Arnold L. Glass, Tingtao Wang, Allyson Fu

**Affiliations:** Rutgers University, 152 Frelinghuysen Road, Piscataway, NJ 08854 USA

**Keywords:** Memory, Visual working and short-term memory, Reaction time methods, Visual working memory

## Abstract

Participants responded whether a single digit was in the immediately preceding digit sequence by pressing one of two keys as rapidly as possible while trying to avoid errors. Each participant performed four different kinds of sessions: Either all of the digits of the study sequence were presented in the same position or the digits were presented in successive positions from left to right. Either the *same* response was assigned to the right key and the *different* response to the left key or vice versa. Response time (RT) was an increasing function of the length of the study sequence. RT was an increasing function of the target’s position in the study sequence when the *different* response was assigned to the right key. When the *same* response was assigned to the right key, RT was a decreasing function of the target’s position in the study sequence when the study sequence had been presented in one location but there was no effect of target position on RT when the study sequence had been presented from left to right. The effects of study sequence length and target position were independent in the three conditions in which there was an effect of target position. Also, RT decreased for targets that had previously appeared as test items but RT increased for lures that had previously appeared as test items. The results confirm a dual-system hypothesis of recognition involving both the perceived recency of the target and the retrieval of the previous context of the target.

Recently, Kang et al. (2023) found that an apparently irrelevant independent variable, response assignment, determined the results of an immediate recognition task in which a study string of four simultaneously presented consonants was followed by a single test consonant on the screen of a laptop computer. Participants were asked to respond as rapidly as possible whether the test consonant was the same as one of the study consonants or different from them all by pressing one of two response keys: the *f* key or *j* key on the laptop. When the *different* response was assigned to the right (*j*) key, RT for responses to targets was an increasing function of the left to right position of the target in the study string of simultaneously presented consonants. Furthermore, RT for lures was longer than RT for targets, so together the resulting target and lure RT *indicated* that a test item was compared from left to right with a representation of the study sequence, and a *different* response was made when a match between the test consonant and a study consonant was not found.

In contrast, when the *same* response was assigned to the right key, RT for responses to targets was not a function of the position of the target in the study string. Rather, target/lure decisions appeared to be made on a holistic judgment of the perceived recency/novelty of the entire test string. Perceived recency of the entire string implied that the test item had appeared in the study sequence, and thus was a target, and perceived novelty implied that the test item had not appeared in the study sequence, and thus was a lure. Consistent with this hypothesis, if a target had previously appeared on an earlier trial as a test item, which presumably increased its perceived recency, RT to identify it as a target was reduced. However, if a lure had previously appeared on an earlier trial as a test item, which presumably decreased its novelty, RT to identify it as a lure was increased. The different effects of a previous presentation as a test item on target versus lure RT found by Kang et al. (2023) were consistent with the effects found by Atkinson and Juola (1973, 1974), Glass (1993), Glass et al. (1974), Kristofferson (1972a, b), and Monsell (1978) for similar immediate visual recognition tasks, in which a study sequence was presented one character at a time rather than presenting an entire study string simultaneously. Also, the effect of response assignment had been found for sequential presentation,

but one had to consult two different reports published a decade apart to discover it. Ashby et al. (1993) had given four participants extensive practice on the study string–test item task for strings of lengths 2 to 5. For all four participants, the *same* response was assigned to the right key and for three of the four participants RT for responses to targets was not an increasing function of the left to right position of the target, consistent with the finding of Kang et al. (2023). Previously, Franklin and Okada (1983) had also investigated the study string–test item task for strings of lengths 2 to 5. For all participants, both response keys were pressed by the right hand and the *different* response was assigned to the right key. RT for *same* responses to targets was an increasing function of the left to right position of the target in the study. This response assignment was identical to one of the conditions of Experiment 3 of Kang et al. (2023), which produced the identical result. Also, Kang et al. (2023) performed control experiments that ruled out the possibility that the Simon effect was relevant to the response assignment × target position interaction.

The discovery of the effect of response assignment on RT: Two entirely different patterns of RT results indicating two entirely different computational processes generating the recognition response was not serendipitous but an a priori prediction of Glass (2019) and consistent with a similar finding by Sinha and Glass (2017).

A large number of studies of animal (Packard, 1999; Packard & McGaugh, 1996; Packard & Teather, 1997), including human (Baumann et al., 2010; Brown et al., & Stern, 2012; Doeller et al., 2008; Hirshhorn et al., 2012; Konishi et al., 2013; Marchette et al., 2011; Wegman et al., 2014; Woolley et al., 2013) navigation, beginning with Packard and McGaugh (1996), found that when exploring a new area, one neural system, including the hippocampus, directs the exploration by distinguishing locations that have just been visited from those that have not been visited, but when traversing a familiar area another neural system, including the caudate nucleus, retrieves a sequence of actions that generate the route: a sequence of left or right turns in response to local features that lead to the goal. The general explanation proposed for these results across these studies is that the mammalian, including the human, brain contains a neural system for way-finding that includes the hippocampus and which controls behavior during exploration and a neural system for route-following that includes the caudate nucleus of the basal ganglia, and which controls behavior when traversing a familiar route.

However, Glass (2019) and Ullman (2004) pointed out that the fact that the different operations of the two distinct neural systems were discovered during a navigation task does not imply that the functions of the two systems are restricted to navigation. Rather, they proposed that the two neural systems discovered in the navigation task performed two general functions in a variety of tasks. What had been identified as only a way-finding system was actually an improvisational system, that directed actions in response to novel tasks and situations, and what had been identified as only a route-following system was a habit system that generated sequences of previously successful actions in response to familiar tasks and situations.

Sinha and Glass (2017) proposed that an immediate recognition task provided evidence of the separate contributions of the improvisational system and the habit system to recognition. When a study string of four simultaneously presented consonants was immediately followed by a test string of four simultaneously presented consonants, a participant had to respond whether the test string was the same or different from the study string by pressing the *f* or *j* key on a laptop keyboard with the left and right index fingers, respectively. When the strings were different, RT was a linearly increasing function of the first left to right difference between the strings, indicating that the strings were compared from left to right until a difference was found. However, *same* RT was shorter than *different* RT, indicating that the *same* response was not based on a left to right comparison of the strings. Sinha and Glass (2017) hypothesized that the *different* responses were the result of left to right sequential comparison of the strings by the habit system but *same* responses were the result of the perceived recency of an immediately repeated string by the improvisational system. Sinha and Glass (2017) recorded fMRI during a replication of the same/different task and found that hippocampal activity consistent with activity recorded during way-finding during *same* responses and caudate activity consistent with activity found during route-following during *different* responses, confirming their prediction that what had been called the way-finding and route-following systems were also responsible for performing an immediate recognition task. To generalize this result to another task, Kang et al. (2024) recorded fMRI when a study string was followed by a *single* test consonant. This time, as mentioned above, when the right key was assigned the *same* response, the test consonant was identified as a target or lure on the basis of its recency/familiarity and hippocampal activity consistent with activity recorded during way-finding was recorded, but when the right key was assigned the *different* response, the test consonant was compared from left to right with the study string and caudate activity consistent with activity recorded during route-following was recorded. Furthermore, both Sinha and Glass (2017) and Kang et al. (2024) found that caudate activation during serial comparison was associated with activation in a different part of the hippocampus than was active during the discrimination between recency and novelty. Together, the behavioral and fMRI results of Sinha and Glass (2017) and Kang et al. (2024) demonstrate that the two neural systems that perform distinct functions in navigation perform the same functions in immediate recognition.

The general purpose of the experiment reported here was to investigate whether the selection of a single neural system performing the immediate recognition task by response assignment would generalize to other immediate recognition tasks in which one or more study characters were followed by a single test character and how the effect of response assignment would interact with the effects of other task variables.

Kang et al. (2024) had presented a four-consonant string. Presumably, the string had been encoded by a sequence of eye-movements from left to right that had successively fixated on each study consonant. Therefore, it seemed likely that if instead of presenting a string, a sequence of consonants was presented from left to right, one at a time, in the positions they would have occupied if they had been presented as part of a string, then the results would be the same. Therefore, the first purpose of the study was to determine whether the same effect of response assignment that had been observed when the characters of a study string were presented simultaneously would also be found when a study sequence was presented one character at a time from left to right in the same positions they would occupy in a simultaneously presented string.

The effect of target position on RT when the right key was assigned the *different* response might depend on how the study sequence was presented. For example, it might only occur when the study sequence was presented from left to right, thus encouraging spatial, left to right, encoding. In this case, when all the members of a study sequence were presented at a central fixation point, there would *not* be effect of target position on RT when the right key was assigned the *different* response. However, this prediction was disconfirmed by the results of two previous studies in which a study sequence was presented at a central fixation point, followed by a test item. Both for digits (Franklin & Okada, 1983) and color patches (Guida et al., 2018), RT increased as a function of target position when the right key was assigned the *different* response. Furthermore, both for digits (Ashby et al., 1993) and color patches (Guida et al., 2018), RT did not increase as a function of target position when the right key was assigned the *same* response. Thus, when the results of three studies, in which the members of a study sequence were presented at a central fixation point, are considered together (Ashby et al., 1993; Franklin & Okada, 1983; Guida et al., 2018), the same interaction between the effects of response assignment and target position that was observed by Kang et al. (2023), when the characters of the study string were presented simultaneously, was found when the study sequence was presented at a central fixation point. Therefore, the second purpose of the study was to confirm that the same effect of response assignment that had been observed when the characters of a study string were presented simultaneously would also be found when a study sequence was presented at a central fixation point. Therefore, the effects of both presentation mode, left or right versus fixed location, and response assignment on RT were included in the design of the experiment reported here.

Kang et al. (2024) did not vary the length of the study string, but both Ashby et al. (1993) and Franklin and Okada (1983) varied the length of the study sequence. Both Ashby et al. (1993) and Franklin and Okada (1983) found that RT was an increasing function of the length of the study sequence, and Franklin and Okada (1983) found independent effects of the length of the study string and the position of the target in the study string on RT. Therefore, the third purpose of this experiment was to determine whether RT would be an increasing function of study sequence length and the effect of study sequence length was independent of the effect of target position, thus replicating the results of Ashby et al. (1993) and Franklin and Okada (1983).

Also, in Sternberg’s (1966) seminal study, using RT to investigate immediate recognition, a sequence of one to six digits was presented at a central fixation point, followed by a test digit. Sternberg found that RT for both targets and lures was a linear function of the length of the study sequence and the slopes of the functions for targets and lures did not differ. If RT was a function of the position of the target in the study sequence, then the slope for targets would have been less than the slope for lures, so the identical slopes implied that there was not an effect of target position in the study sequence on RT. Sternberg (2016) acknowledged that his results were only replicated when his exact task was replicated. For example, as mentioned above, in other versions of the immediate recognition task an effect of the position of the target in the study sequence on target RT was found. Sternberg did not include the effect of response assignment in his analysis. Furthermore, the review articles describing the results of the many variants of the original task reviewed by Sternberg (1975) and Sternberg (2016) did not report including response assignment in the analysis of the results. Therefore, the fourth purpose of the experiment was to investigate whether including response assignment in the analysis of the results of Sternberg’s (1966) task would provide evidence of an effect of response assignment that would reconcile his results with the results of other immediate recognition tasks. Therefore, the study sequences were composed of digits, and targets and lures were digits.

Participants performed a within-subject experiment with four conditions. In half of the sessions the study sequence was presented in a single location, which was the task used by Sternberg (1966), and in half of the sessions, the study sequence was presented from left to right. Within each study sequence presentation mode, for half of the sessions, the *same* response was assigned to the right key, and for half of the sessions, the *different* response was assigned to the right key.

## Method

The experiment received the approval of the Rutgers University Institutional Review Board under the identifier Pro20170000798.

### Design and materials

The experiment was composed of four sessions performed over a 2-week period. An experimental session was composed of a total of 168 trials. The number of study sequences of each length were distributed among the trials as follows:

**Table:** 

Study sequence	# of trials
**length**	
One	8
Two	16
Three	24
Four	32
Five	40
Six	48

For each study sequence length, on half of the trials the test item was drawn from the study sequence. When the test item was drawn from the study sequence, it was drawn equally often from each position in the study sequence.

The task was performed on a laptop computer. Study sequences and test items were presented on the laptop screen. The *f* and *j* keys on the keyboard were the left and right response keys, respectively.

There were four kinds of experimental sessions. On two of the experimental sessions, the location of the study items was fixed. All of the study items were presented in the center of the screen at the fixation point. For the other two experimental sessions, each study item in the sequence was presented to the right of its predecessor. For a study sequence length of 1, the single study item was presented at the fixation point at the center of the display. Each digit of longer sequences was presented in a separate location from left to right across the display so that for study sequence lengths of 3 and 5, the middle item was presented at the fixation point, and for study sequence lengths of 2, 4, and 6, the first half of the sequence was presented before the fixation point and the second half of the sequence was presented after the fixation point. The locations of the study items were where the digits would appear if they comprised a single number.

For one of the two sessions, when the entire study sequence was presented at the fixation point, the right key was assigned the *same* response (test item in study sequence; i.e., target) and the left key was assigned the *different* response (test item not in study sequence; i.e., lure). For the other session, the right key was assigned the *different* response, and the left key was assigned the *same* response.

Similarly, for one of the two sessions, when the study sequence was presented from left to right, the right key was assigned the *same* response and the left key is assigned the *different* response. For the other session, the right key was assigned the *different* response and the left key was assigned the *same* response.

All participants performed all four kinds of sessions, performing two sessions with the same response assignment on Monday and Wednesday of the same week. Participants were assigned to one of four groups that performed the sessions in different orders over a 4-week period.

### Procedure

When a student was recruited to participate in the experiment, the student was told that they would have to respond as rapidly as possible but would also have to be highly accurate so that they would have to pay close attention to the task. The experiment was performed on a computer laptop. The study sequence and test item were presented on the laptop screen, and responses were made by pressing the *f* and *j* keys on the laptop keyboard.

Each experimental session began with a practice session of 24 trials. For the practice trials, the study sequence and test item were randomly drawn from the pool of 20 English consonants. Otherwise, the procedure was the same as for the experimental trials. There were four study sequences each of from 1 to 6 consonants. On two trials for a study sequence of each length, the test item was in the study sequence.

On each experimental trial, a different study sequence of from 1 to 6 digits was presented, randomly selected from the pool of 10 digits. Trials were self-paced. A trial began with the word “Ready,” which remained on the screen until a response was made, which blanked the screen. One second later the first digit of the study sequence appeared and successive digits appeared at the rate of one digit every 1.2 s. Two s after the offset of the final digit, an asterisk appeared at the fixation point for 1.2 s, which was replaced by a test digit. The task was to respond as to whether the test digit was in the study sequence by pressing one of two response keys as rapidly as possible without making an error. The *f* and *j* keys on the laptop keyboard were the left and right keys, respectively. The left index finger was placed on the *f* key and the right index finger was placed on the *j* key.

The test item remained on the screen until a response was made. The screen remained blank for 1 s, and then the “Ready” signal appeared again. Furthermore, to encourage participants to take breaks if they were becoming bored or fatigued, the 168 trials were divided into 42 trial blocks by three rest periods. A rest period began immediately after a response to a test item. It would last for up to 2 min before the next “Ready” signal appeared. However, a participant could end the rest period at any time by pressing the space bar.

### Participants

Each participant was a student in an Advanced Topics class. Experimental participation was a class assignment. There were 19 participants (13 women).

## Results

**Fig. 1:**
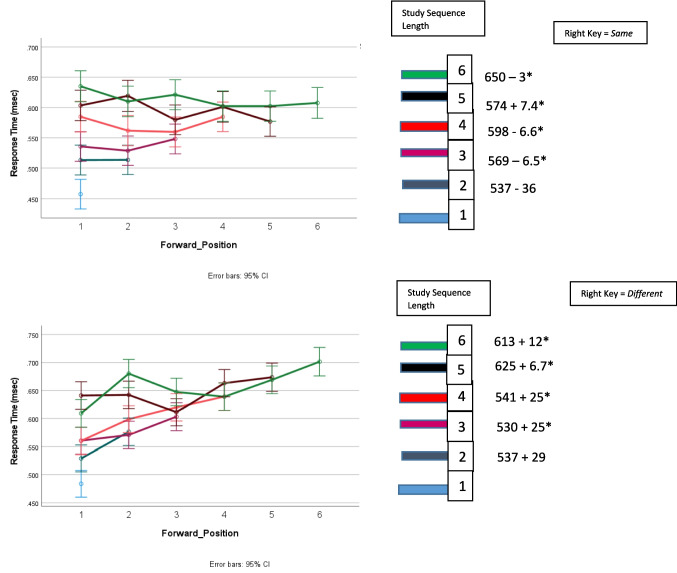

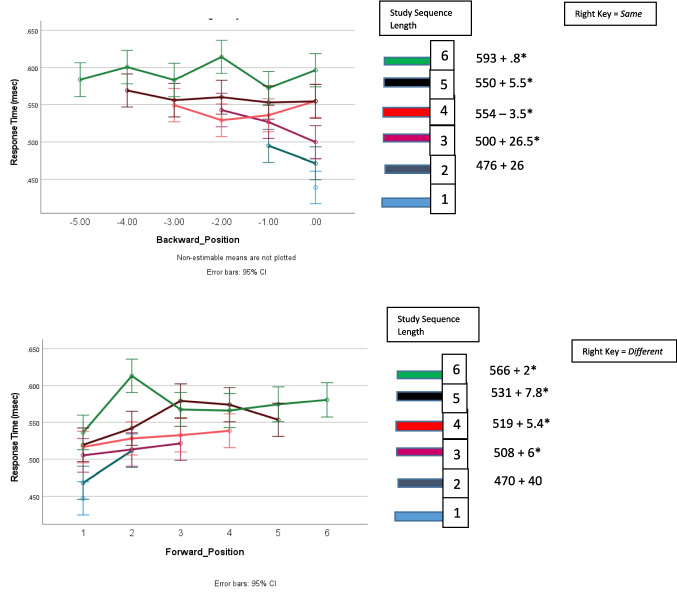
The effects of response assignment, study position of target, and study sequence length on RT for same responses to targets when the study sequence presentation was from left to right (top panels) and when study sequence presentation is at a central fixed point (bottom panels). * indicates significant at < .02. (Color figure online)

**Fig. 2:**
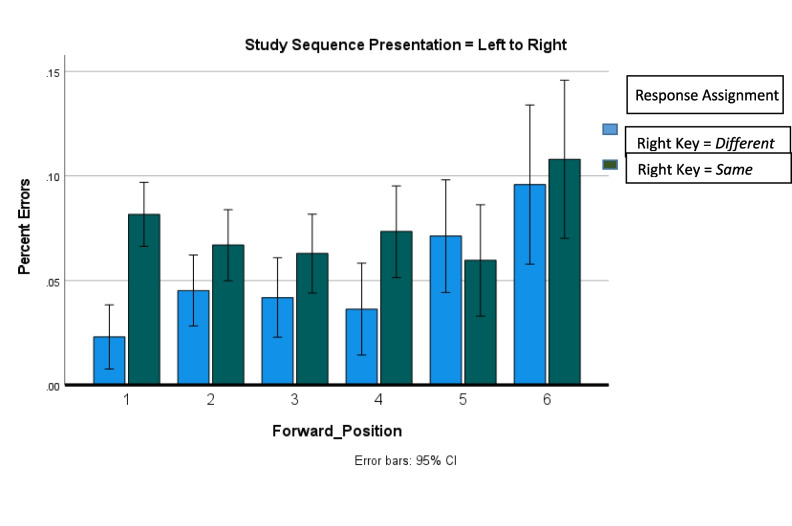
The effects of response assignment and study position of target on percentage error for responses to targets when the study sequence presentation was from left to right

**Fig. 3:**
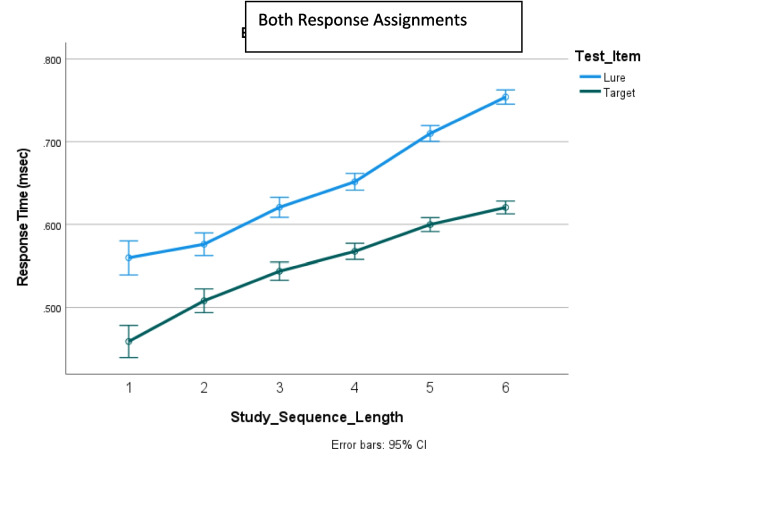

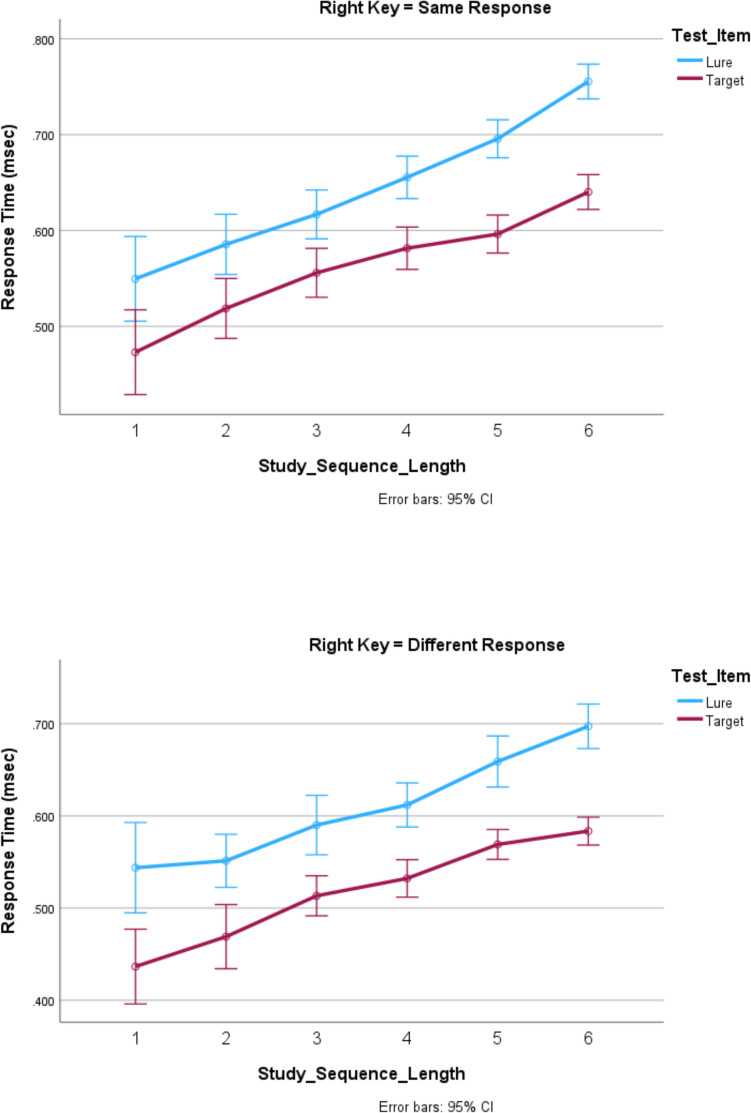
The effect of study sequence length and test item type on RT for correct responses when the study sequence was presented at a central fixation point for eight participants with mean error rate of 1.6%. (Color figure online)

**Fig. 4:**
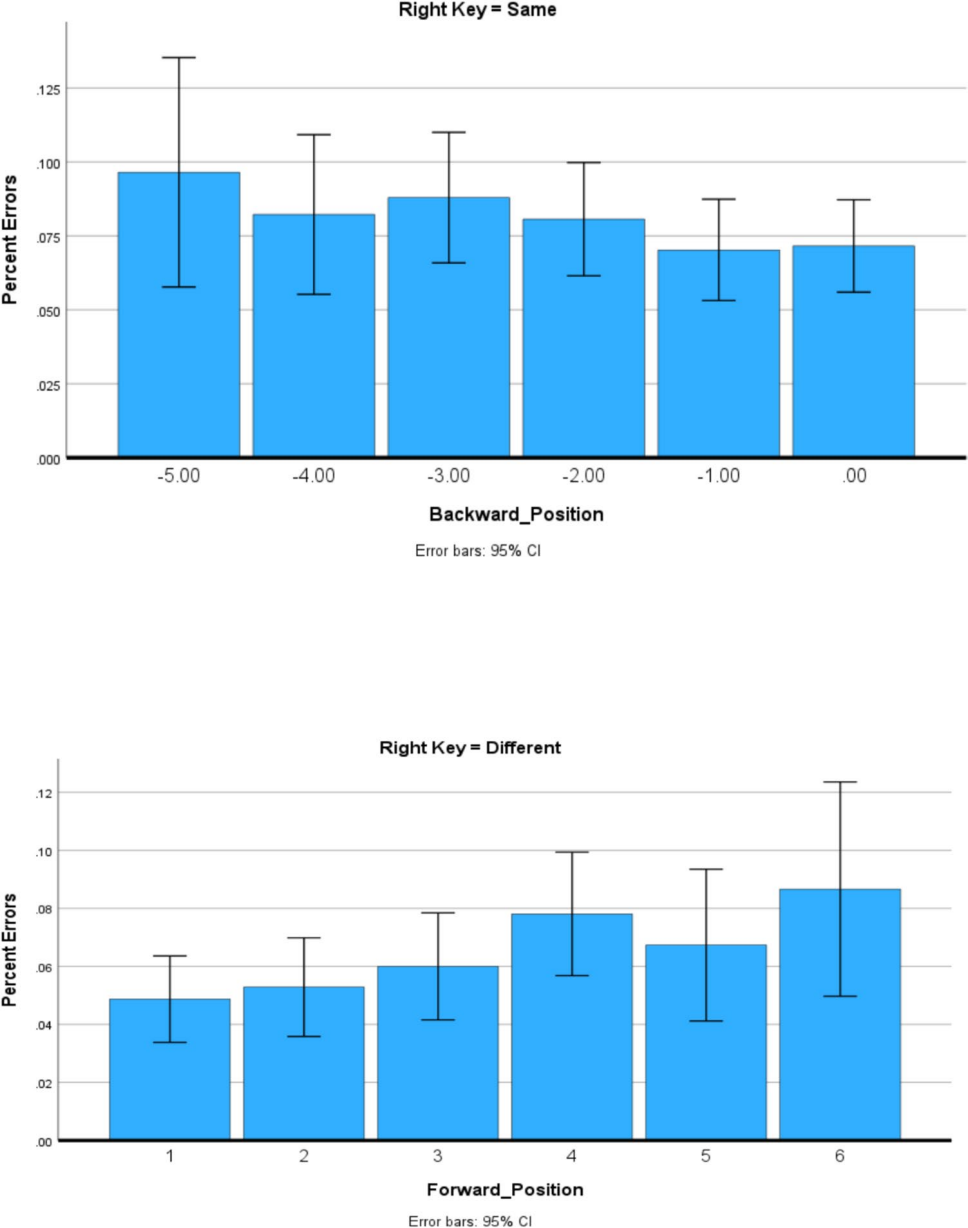
The effects of response assignment and study position of target on percentage errors for responses to targets when study sequence presentation is at a central fixed point

**Fig. 5:**
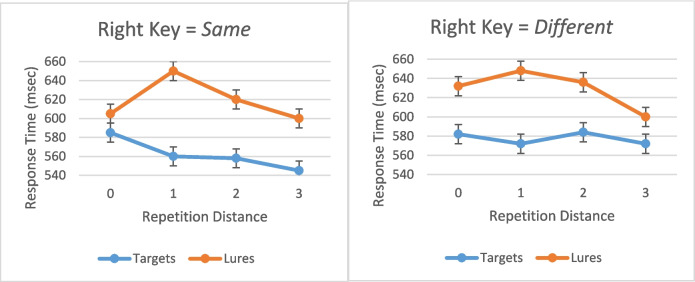
The effect of repetition distance of the test digit on response time for responses to targets and lures. (Color figure online)

There were 0.65% of the responses to targets and 0.46% of the responses to lures that were more than three standard deviations from mean RT. These responses were eliminated from all analyses. Of the remaining responses, 6% of the responses to targets and 6% of the responses to lures were errors. These responses were eliminated from all analyses of RT. All analyses were univariate analyses for the general linear model, for which *participants* was the random variable. Effects significant at the *p* = 0.05 level are reported. Reporting details of the *F* tests for all significant results in the text made it unwieldy, so for the sake of the clarity and conciseness of the text, the details of the *F* tests are shown in Table 1. Table 1 also shows the figure or figures showing the results analyzed by the *F* test. For the analysis of the results shown in Figs. 1, 2, 3, 4 and 5, all main effects and interactions significant at the *p* = 0.05 level are reported in Table 1. All of the analyses were univariate analyses for the general linear model provided by the Statistical Package for the Social Sciences (SPSS). Participants was always the random effect in the analysis. The data plots plotted in Figs. 1 through 4, the data points plotted are the estimated marginal means for the univariate analysis of the general model by SPSS.

**Table 1:** F-tests for results

Independent variables	*F* tes	$\eta_{p}^{2}$
Figure 1** (target RT)**		
Study sequence length	*F*_5,11667_ = 119.3, *p* < .001	.049
Target position	*F*_5,11667_ = 3.3, *p* = .005	.001
Study sequence presentation × response assignment	*F*_1,11667_ = 75.5, *p* < .001	.006
Response assignment × study sequence length	*F*_5,11667_ = 2.6, *p* = .024	.001
Response assignment × target position	*F*_5,11667_ = 10.3, *p* < .001	.004
Study sequence presentation × response assignment × target position	*F*_5,11667_ = 2.2, *p* = .053	.002
Study sequence presentation × study sequence length × target position	*F*_10,11667_ = 2.8, *p* = .002	.002
Figure 2** and **Fig. 4 (error rates)		
Response assignment × target position	*F*_5,12412_ = 12.2, *p* = .05	.001
Figure 3** (RT)**		
Study sequence length	*F*_5,11713_ = 165.5, *p* < .001	.066
Test item	*F*_1,11713_ = 465.3, *p* < .001	.038
Study sequence length x test item	*F*_5,35_ = 4.7, *p* = .002	.403
Figure 5** (RT)**		
Repetition distance	*F*_3,23583_ = 9.8, *p* < .001	.001
Response assignment × repetition distance	*F*_3,23583_ = 2.59, *p* = .051	< .001
Test item × repetition distance	*F*_3,23583_ = 10.1, *p* < .001	.001
Response assignment × repetition distance × test item	*F*_3,23583_ = 4.35, *p* = .005	.001

First, the results when the study sequence was presented from left to right so the task was similar to that of Kang et al. (2024) are presented; second, the results when the study sequence as presented at a central fixation point so the task was similar to Sternberg (1966) are presented; and third, results general to all four types of study sessions are presented.

### When the study sequence was presented from left to right the results of Kang et al. (2024) were replicated and elaborated

As shown in Fig. 1, for study sequence lengths of one through four, as Kang et al. (2024) found, RT for *same* responses to targets increased as a function of the position of the target in the study sequence when the *different* response was assigned to the right key but there was no effect of the target’s position in the study sequence position when the *same* response was assigned to the right key. In addition, Fig. 1 shows that RT also increased as a function of the length of the study sequence for both response assignments and the effect of study-sequence length was independent of the effect target position.

Figure 1 includes the results for study sequences of lengths five and six that were presented from left to right. Figure 1 shows that RT also increased as a function of the length of the study sequence for both response assignments for study-sequence lengths of one to six, and there was an increasing function of a target of Positions 1–4 when the right key was assigned the *different* response. There was not an interaction between the effects of study-sequence length and target position.

Figure 2 shows the relationship between target position and error rate. Figure 2 shows that target error rate was lower when the right key was assigned the *different* response than when the right key was assigned the *same* response for target Positions 1–4.

Therefore, as shown in Fig. 1 and in Fig. 2, the effects of response assignment on target RT and target error for Positions 1–4 are not observed for positions five and six. The implications of these behavioral differences will be considered in the Discussion section.

### When the study sequence was presented at a central fixation point, key results of Sternberg (1966) were replicated and elaborated

Figure 3 shows the results for the presentation of the study sequence in a fixed location, which is the same procedure used in Sternberg’s (1966) varied set procedure. Sternberg (2016) noted that subsequent studies employing variants of his procedure reported higher error rates and smaller slopes. Therefore, to make our results directly comparable, the RT results for the eight participants with the lowest error rates are shown in Fig. 3. The error rate was 1.6% for the eight participants, compared with 1.3% for the eight participants that Sternberg included in his analysis.

Sternberg (1966) found that when collapsed over response assignment, RT was a linear function of study sequence length for both targets and lures. Sternberg (1966) found the slope of the study-sequence-length function was 38 ms for both targets and lures for the eight participants in his experiment. As shown in Fig. 3, our slope for targets was 34 ms for targets and 39 ms for lures.

However, Sternberg reported a common *y*-intercept for targets and lures at 397 ms. As shown in Fig. 3, we found an intercept of 445 ms for targets and 560 ms for lures.

Sternberg (1966) did not find an effect of the target’s position in the study sequence on target RT, but Sternberg did not include response assignment in the analysis. When response assignment was not included in the analysis of the results shown in Fig. 3, there was not an effect of target position on target RT, *F*_5,35_ = 0.693, *p* = 0.632, consistent with the finding of Sternberg (1966).

When response assignment and target position were included in the analysis along with study sequence length, two new effects emerged. First, consistent with results for the left to right presentation of the study sequence, there were independent effects of both study sequence length and target position. Also, consistent with results for the left to right presentation of the study sequence, when the study sequence was presented at a central fixation point and the right key was assigned the different response, target RT was an increasing function of the ordinal position of the test item in the study sequence, as shown in the bottom panel of Fig. 1. However, when the study sequence was presented at a central fixation point and the right key was assigned the same response, target RT was a *decreasing* function of the ordinal position of the test item in the study sequence, as shown in the third panel of Fig. 1. There was a significant interaction between response assignment and target position, *F*_5,35_ = 2.5, *p* = 0.049, for the eight participants with the lowest error rate, as well as for all 19 participants in the experiment, as shown in Table 1.

In order to accurately plot the interaction between the effects of response assignment and target position on target RT in the bottom panels of Fig. 1, target position is computed in different ways for the different response assignments. For right key = *same*, the backward position of a target in the study sequence was counted from the end of the sequence so the last digit in the study sequence was in Position 0. The third panel of Fig. 1 shows that when the right key = *same*, target RT was an increasing function of its *backward* position in the study sequence for sequences of Lengths 1–3 and flat for longer sequences. For right key = *different*, the *forward* position of a target in the study sequence was counted from the beginning of the sequence so the first study digit presented was in Position 1. The bottom panel of Fig. 1 shows that when the right key = *different*, target RT was an increasing function of its *forward* position in the study sequence for sequences of lengths one through four and flat for longer sequences.

Forward position is *not* a linear transform of backward position and vice versa. They are two different ways of plotting the data that may reveal different effects on it. For forward position, the targets in the first position of the sequence for all sequence lengths (1–6) are assigned Position 1 *and therefore contribute values to a single data point in the figure*. Targets in the last position of sequences of Lengths 1–6 are assigned Positions 1–6 *and therefore contribute values to different data points in the sequence*. For backward position, the targets in the last position of the sequence are all assigned Position 0 *and* *so contribute values to a single data point in the figure,* and the targets in the first position of sequences of Lengths 1–6 are assigned positions 0 through − 5 *and* *so contribute values to different data points in the sequence*. The interaction between response assignment and target position was significant regardless of whether forward position or backward position was used in the analysis. Plotting the effect of target position as an effect of forward position in the left panel would have obscured the effect of backward position that was the cause of the interaction between target position and response assignment. Therefore, the effect of backward position is shown in the bottom panel of Fig. 1. As mentioned above, when response assignment was not included in the analysis, the opposing increases in target RT as a function of backward versus forward target position cancelled each other out so there was not a significant effect of target position on target RT.

As Table 1 reports and Fig. 4 shows, when study sequence presentation was at a central fixation point the interaction of the effects of response assignment and target position on error rate was the same as the interaction for RT. Error rate was an increasing function of backward position of the target when the right key was assigned the *same* response, and error rate was an increasing function of forward position of the target when the right key was assigned the *different* response.

### The effect of recency/novelty of test item on RT

The number of trials back (NTB) that the digit used as the test item last appeared as a study or test item was counted for the test item that appeared on each trial of a session. The use of a digit as a study item had no effect on RT when it was subsequently used as test item, but the use of a digit as a test item did have an effect on RT when it was subsequently used as a test item, as shown in Fig. 5. NTB was divided into four levels, labelled from 0 to 3, such that NTB = 0 when the digit used as the test item had not previously appeared in the session, NTB = *n*, *n* = 1 to 2, when the digit used as the test item had last appeared on the *n*th previous trial back, and NTB = 3, when the digit used as the test item had last appeared in the third previous trial back or an earlier trial. Presentation, response assignment, test item, repetition distance (number of trials between successive appearances of a digit as a test item) and consistency (whether the digit had previously appeared only as a target, only as a lure, or at least once as each) were the independent variables of a univariate analysis of RT. As reported in Table 1 and shown in Fig. 5, there was an interaction between repetition distance and response assignment. RT increased for a lure that had been the test digit on the immediately previous trial but not for longer repetition distances. This effect was greater when the response key was assigned the same response. Also, RT decreased for a target that had previously appeared as a test digit regardless of repetition distance when the right response key was assigned the *same* response but not when the right response key was assigned the *different* response.

## Discussion

The results of this study have both theoretical and methodological implications.

### Theoretical implications

The RT results shown in Fig. 1 and the accuracy results of Fig. 2 and Fig. 4 are consistent with the assumption that assigning the *different* response to the right key elicits comparison of the study item with the study sequence for study sequences of Lengths 1–4, but for study sequences of Lengths 5 and 6 a same/different decision is based on the perceived recency/novelty of the test item.

This finding is explained by the continuous, aggregated, accumulation model of recognition (Kang et al. 2024). This model assumes that recency/novelty information is combined with information generated from a comparison between the test item and the study context and when the amount of information exceeds a boundary that collapses over time, a response is made. When the *same* response is assigned to the right key, then recency/novelty information is available first and often immediately exceeds the response boundary before information from the comparison of the study context accumulates. In contrast, when the *different* response is assigned to the right key, the comparison of the test item with the study sequence begins before recency/novelty information is available.

A collapsing boundary determines the amount of information that must accumulate for a recognition decision. The boundary collapses to zero so that after a brief period of time a response is made on the basis of whatever information is available. The rate at which the boundary collapses is under voluntary control. Participants are instructed to respond as rapidly as possible without making an error. However, delaying a response until all available information had accumulated would result in many long RTs. Therefore, participants may trade accuracy for speed and adopt a response boundary that is reached before a five or six item sequence can be completely searched at test, so the information controlling the response to the test item for five and six item study sequences is predominantly recency/novelty information, resulting in shorter RTs but more errors. Consequently, when the right key is assigned the *different* response, there is no effect of target position for study sequence lengths of five and six. Furthermore, when the study sequence is presented from the left to right, for target Positions 1–4, the error rate was lower when the right key was assigned the *different* response than when the right key was assigned the *same* response, indicating that retrieving the study context resulted in a more accurate response than only assessing recency; however, for target Positions 5 and 6, the error rate was not significantly different when the right key was assigned the *different* response than when the right key was assigned the *same* response, indicating that the recency information was the primary influence for both response assignments.

### Study sequence presentation at a central fixation point

Sternberg (1966) apparently did not find an effect of target position when the study sequence was presented at a central fixation point. This finding was replicated when Sternberg’s analysis, which did not include response assignment, was repeated. However, when response assignment was included in the analysis a significant interaction in the effects of target position and response assignment on RT was found: Target RT was an increasing function of the ordinal position of the test item in the study sequence when the right key was assigned the *different* response but target RT was a decreasing function of the ordinal position of the test item in the study sequence when the right key was assigned the *same* response. The reason there was no effect of target position when response assignment was not included in the analysis is that the opposite effects for right key = *different* and right key = *same* cancelled each other out.

It is possible that the opposing effects of backward and forward target position cancelled each other out also existed in Sternberg’s (1966) data, but it is also possible that there was no effect of target position on RT regardless of response assignment in Sternberg’s (1966) experiment because Sternberg’s (1966) method was not precisely replicated and there was another difference between the results reported here and those of Sternberg (1966). As shown in Fig. 5, the previous appearance of a target or a lure as a test item decreased RT for targets and increased RT for lures. Therefore, when RT was plotted as a function of target position both the intercepts and slopes of the function were greater for lures than for targets. However, Sternberg had participants recall the study sequence after every recognition response. Perhaps, in order to recall the study sequence accurately, the study sequence was encoded in a way that provided parallel access to all members of the sequence at test, so there was no effect of target position in Sternberg’s (1966) experiment. Or, perhaps in order to recall the study sequence accurately, the study sequence was encoded in a way that had the effect of increasing the perceived difference between the perceived recency of a target and the perceived novelty of a lure, thus reducing the difference in the study-sequence-length RT functions between the intercepts for targets and the intercepts for lures, while leaving the effects of target position intact. Therefore, the next experiment that must be done is a more exact replication of the method of Sternberg (1966) in which participants report the study sequence after every recognition response.

Regardless of the results of a precise Sternberg (1966) replication, the results reported here provide additional evidence of the existence of the improvisational and habit systems. Again, assigning the *different* response to the right key resulted in self-terminating left to right comparison of the test digit with the study sequence. However, there was also an effect of presentation mode. Perhaps when the study string is presented at a central fixation point and the right key is assigned the *same* response, verbal encoding of the study sequence is elicited and this results in the backward target position effect, which is identical with a verbal recency effect. In fact, Conrad (1964) found verbal recoding of a visual consonant sequence and this result implies that there will also be verbal recoding of a visual digit sequence.

Sternberg (1969) pointed out that if two different variables influence the durations of two different sequential stages of the recognition process, such as the encoding stage and the response stage, then their separate effects on observed RT will be independent and additive. Therefore, the independent effects of sequence length and target position imply that the factors affect different stages of the recognition task. In fact, Checkosky (1971) and Checkosky and Baboorian (1972) found evidence that after the test item was presented, first the study sequence was serially generated and then compared with the test item in order for a recognition judgment to be made (see also Holyoak et al., 1976). Therefore, the effect of study-sequence length on RT may be the result of the effect of study-sequence length on a study-sequence generation stage that after the test item is presented but prior to the comparison of the study-sequence with the test item.

Furthermore, the finding that an appearance of a digit as a test item influences its perceived recency, hence RT when the digit is again subsequently used as a test item, is consistent with the findings of Atkinson and Juola (1973, 1974) Glass (1993), Glass et al. (1974), Kristofferson, (1972a, b), and Monsell (1978) for similar tasks. This finding indicates that a comparison between the study sequence and test item may not be necessary in order for an accurate recognition response to be made.

### Methodological Implications

Details matter. In an analysis of RT, all independent variables of the experimental design that can be counterbalanced, such as response assignment, should be counterbalanced. All independent variables, including those included only for counterbalancing purposes, should be included in the analysis. In this experiment, including response assignment in the analysis revealed an effect that would otherwise have remained occult.

The contrast methodology, comparing response assignments and presentation modes, provides the ideal experimental design for revealing the effects of distinct functional systems whose effects on the response are usually aggregated. Furthermore, through the recording of neural images (Kang et al., 2024; Sinha & Glass, 2017) or event related potentials the distinct functional neural systems producing the behavioral effects can be identified.

### Significance of results

Gazzaniga et al. (1962) found evidence in the results of a few unusual tasks that the same target was processed independently by the right and left hemispheres of a single person and the results were replicated in investigations of about a dozen patients whose brains had suffered damage from life-threatening epilepsy and then had the corpus callosum severed. Nevertheless, these results have been highly influential.

The results of Sinha and Glass (2017), Kang et al. (2024), and of the experiment reported here show that such a complete dissociation of cognitive systems can be achieved with ordinary college students through the effect of response assignment in a task for which RT is the dependent measure. The results of fMRI in the Sinha and Glass (2017) and Kang et al. (2024) studies confirmed that two entirely neural systems, including subcortical structures, controlled the responses, depending on the response assignment. Analyzing the effects of such task factors as presentation mode and response assignment is an extremely effective tool for identifying the distinct roles of the improvisational system and the habit system in human cognition. Finally, Kang et al. (2024) have fit a continuous, aggregated, accumulation, drift–diffusion model that combines perceived recency/novelty with recollected details of the study context to the results of a recognition task. The model provides a framework for the further development of a dual system explanation of recognition.

If two entirely different functional neural systems can perform an immediate visual recognition task, then one can be certain that the two systems perform different functional roles in the myriad of more complex tasks that include visual recognition. One example is tasks that investigate whether numerical magnitude is mentally encoded along a spatial number line. In an experiment originally intended to investigate how numbers are encoded, participants were first given a criterion number followed by a sequence of test numbers. The task was to respond to each test number when it appeared as rapidly as possible while avoiding an error of whether the test number was less than or greater than the criterion number by pressing one of two response keys. There was a congruity effect on RT: RT was shorter when the “less than” response was assigned to the left key and “greater than” response was assigned to the right key than when the “less than” response was assigned to the right key and “greater than” response was assigned to the left key. The congruity effect between the magnitude of the test number and the position of the response key is called the Spatial Numerical Association of Response Codes (SNARC) effect (Dehaene et al., 1993).

Van Dijck and Fias (2011) hypothesized that the SNARC effect occurred because a working-memory spatial representation of the task relevant fragment of the number line in which the number line was represented in ascending order from left to right was encoded and consulted when someone performed the magnitude comparison task. To demonstrate the role of a working-memory spatial representation, in Experiment 1 of their report they had participants perform a task in which a study sequence of five randomly selected and ordered numbers was presented at a central fixation point and followed by 20 test numbers within the range. When the test number was a member of the study sequence, the participant had to press one of two response keys to indicate that the number was equal to or less than five, or press the other response key to indicate that the number was equal to or greater than six. Consistent with the working memory hypothesis, there was a congruity effect between the left/right position of the response key and the left to right position of the test number in the study sequence, rather than between the left/right position of the response key and the magnitude of the test number. The congruity effect between the position of the test number in the study sequence and the position of the response key is called the Spatial Positional Association of Response Codes (SPoARC) effect.

Van Dijck and Fias (2011) interpreted their result as supporting their hypothesis that the SNARC effect was the result of the congruity or incongruity between the position of the response key and the position of the test item in the study sequence. However, notice that the task employed by Van Dijck and Fias (2011) included two subtasks. When the test number appeared, the first subtask was to determine whether the test number was in the study sequence. If the test number was in the study sequence, then the second subtask was to respond whether the test number was equal to or less than five or the test number was equal to or greater than six. The two-task procedure raises the issue of whether the SNARC effect is caused by the first subtask: determining whether the test number was in the study sequence or the second subtask: responding whether the test number was equal to or less than five or the test number was equal to or greater than six.

The results of the experiment reported here indicate that the SNARC effect occurs during the first subtask: determining whether the test number was a member of the study sequence. The results of the experiment reported here were that when the right response key indicated that the test number was in the study sequence, then the RT to identify the test item as a member of the study sequence was an increasing function of the first to last position of the test item in the study sequence. But when the left response key indicated that the test number was in the study sequence, then the RT to identify the test item as a member of the study sequence was an increasing function of the last to first position of the test item in the study sequence. In Van Dijck and Fias’s (2011) task, the right response key indicated that the test number was in the study sequence and the test number was equal to or greater than six. The results of the experiment reported here were that when the right response key indicated that the test number was in the study sequence RT was an increasing function of the last to first position of the test item in the study sequence. In Van Dijck and Fias’s (2011) task, the left response key indicated that the test number was in the study sequence and the test number was equal to or less than five. The results of the experiment reported here were that when the left response key indicated that the test number was in the study sequence RT was an increasing function of the first to last position of the test item in the study sequence. Therefore, the results of Van Dijck and Fias’s (2011) first subtask, determining whether the test number was in the study sequence, produced the SNARC effect in Van Dijck and Fias’s (2011) experiment.

Notice also that when Van Dijck and Fias’s (2011) task is performed it may be the case that the second subtask is performed before the first subtask. First, a participant determines whether the test number is less than or greater than 5.5. Then, the participant determines whether the test item was in the study sequence because only in that case is a response made. Therefore, if the test item is less than 5.5 so the left key must be pressed, then the study sequence is searched from first (left) to last (right). If the test item is greater than 5.5 so the right key must be pressed, then the study sequence is searched from last (right) to first (left).

In summary, the finding of the experiment reported here explains Van Dijck and Fias’s (2011) finding that RT is shorter when the position of the response key is congruent with the position of the test item in the study sequence. The position of the response key determines the order in which the study sequence is searched. Consequently, the left key both implies that the test number is near the beginning of the study sequence and causes the study sequence to be searched from the beginning of the study sequence and the right key both implies that the test number is near the end of the study sequence and causes the study sequence to be searched from the end of the study sequence.

Van Dijck and Fias (2011) demonstrated the generality of the effect of congruence between response key position study sequence position of test beyond numbers in Experiment 2 by obtaining the same congruity effect for fruit/vegetable sequences and a criterion test of whether the test item was a fruit or vegetable.

The results of the experiment reported here also explains the classic SNARC effect under Van Dijck and Fias’s (2011) assumption that in order to perform the task, integers in a range including the criterion number become active in working memory, hence the range is effectively equivalent to a study sequence in the experiment reported here and in Van Dijck and Fias’s (2011) Experiment 1. When a test number is presented, the range is scanned until either the test number or the criterion number is found. When the test number is near the beginning of the range, a left key response is tentatively prepared (but not executed), and the range is searched from the beginning until either the test number or criterion number is found. If the test number is found, then the left key is pressed immediately, but if the criterion number is found, then the prepared response of the left key is inhibited and the right key is pressed. When the test number is near the end of the range, a right key response is tentatively prepared (but not executed), and the range is searched from the end until either the test number or criterion number is found. If the test number is found, then the right key is pressed immediately, but if the criterion number is found, then the prepared response of the right key is inhibited and the right key is pressed.

Finally, Van Dijck and Fias (2011) demonstrated the generality of the effect of congruence between response key position study sequence position of test beyond numbers in Experiment 2 by obtaining the same congruity effect for fruit/vegetable sequences and a criterion test of whether the test item was a fruit or vegetable. This finding demonstrates that the effect of response assignment is not material-specific but a more general effect determined by characteristics of the recognition task, which is consistent with the explanation proposed here.

## Data Availability

The data are currently available from the first author and will be placed in a repository when the report is accepted for publication. The experiment is not preregistered.

## Code availability

Coded in PsychoPy, which is publicly available.
